# Preparation of Thermoresponsive Polymer Nanogels of Oligo(Ethylene Glycol) Diacrylate-Methacrylic Acid and Their Property Characterization

**DOI:** 10.1186/s11671-018-2610-6

**Published:** 2018-07-13

**Authors:** Hongyan Cao, Fenghao Guo, Zhiyong Chen, Xiang Zheng Kong

**Affiliations:** College of Chemistry and Chemical Engineering, University of Jinan, Jinan, 250022 China

**Keywords:** Oligo(ethylene glycol) diacrylate, Methacrylic acid, Crosslinking, Thermoresponsive, Volume phase transition

## Abstract

Stimuli-responsive polymers have received growing attention in recent years owing to their wide applications in diverse fields. A novel stimuli-responsive polymer, based on oligo(ethylene glycol) diacrylate (OEGDA) and methacrylic acid (MAA), P(OEGDA-MAA), is prepared by precipitation polymerization and is shown to have a LCST-type VPTT (volume phase transition temperature) at 33 °C in water and a UCST-type VPTT at 43 °C in ethanol, all at concentration of 1 mg/mL. Both VPTTs are strongly concentration and pH dependent, providing an easy way to tune the phase transition temperature. The polymer is characterized with regard to its composition and its morphology in water and in ethanol at different concentration. The two transitions are studied and interpreted based on the results. This work provides a novel way for the preparation of a new type of stimuli-responsive polymer with great potential for different applications, particularly those in biomedical areas because PEG-based stimuli-responsive polymers are known to be nontoxic and non-immunogenic.

**Electronic supplementary material:**

The online version of this article (10.1186/s11671-018-2610-6) contains supplementary material, which is available to authorized users.

## Background

Stimuli-responsive polymers are able to respond to external stimuli with considerable change in their physicochemical properties [1–3]. A great variety of their applications, including actuators, drug delivery, gene transfer, and materials separation, have been developed [4–7] thanks to this responsiveness. Among the common external stimuli, such as temperature, pH, ionic strength, electric field, and ultrasound, polymers responsive to temperature change, namely thermosensitive polymers, have received great attention for decades [1–9], of which the polymers with a volume phase transition temperature (VPTT) or a lower critical solution temperature (LCST), for no-crosslinked soluble polymers, have been by far the most widely studied. This is particularly true for P(*N*-isopropylacrylamide), PNIPAM, a thermosensitive polymer extensively studied for its potential biomedical applications [2, 9–12] due to its VPTT in water around 32 °C, close to the human body temperature.

It is well accepted that PNIPAM-based materials are of discernable hysteresis, have strong hydrogen-bonding interactions with proteins, and produce low-molecular weight amines during hydrolysis. All these properties have limited their applications in the biotechnology field [13]. Recently, a new family of polymers (PEG) based on oligo(ethylene glycol) (OEG) with thermoresponsive properties has been developed [14–17]. However, PEG in water is only thermoresponsive at elevated temperature and under pressure [18], which makes PEG unsuitable for many applications. To widen its applications as thermoresponsive materials, the common practice is to attach functional groups at one or both of its OEG terminals, making an OEG macromonomer, which is then polymerized through different processes, including for instance anionic [19], cationic [20], group transfer polymerization [21, 22]; conventional free radical polymerization [23]; a variety of free radical-based living polymerizations, including ATRP [24], RAFT [25, 26], and NMP [27]; and diverse other processes of polymerizations [28, 29]. It is now well known that VPTT or LCST of the OEG-based polymers can be adjusted by changing the experimental conditions of their syntheses, in order to change their structures, including for instance the molecular weight, the architecture and the length of EG segment, the ratio and the structure of the comonomers, and the nature of the end groups. All these have been well presented in recent reviews [1, 17].

It is notable that an absolute majority of the reported studies on OEG-based responsive polymers has been prepared with one end of the OEG monomer functionalized by (meth)acrylate while the other end is terminated by an ether. The polymers thus prepared are therefore consisted of a backbone of the (meth)acrylic segments with the OEG chains as the pendant groups, which makes the OEG polymer a comb-like chain. Another common feature for the OEG-based polymers reported up to date is that these polymers have been prepared mostly by polymerization of one single OEG macromonomer [19] or copolymerization of two or more OEG macromonomers of different OEG length or structure [14–16], not crosslinked in either case. In contrast to this background, a novel type of OEG-based polymer, P(OEGDA-MAA), is prepared in this work by copolymerization of oligo(ethylene glycol) diacrylate (OEGDA) with methacrylic acid (MAA), through precipitation polymerization in water. The polymer thus formed is therefore crosslinked because of the diacrylate structure and is shown to be thermoresponsive with a VPTT in water, which is similar to the commonly reported LCST-type phase transition, whereas in ethanol this polymer demonstrates a VPTT of UCST-type. Both VPTTs are closely concentration dependent. These phase transitions are characterized with regard to polymer composition and morphology in dispersion at different concentration. This work provides therefore a novel type of stimuli-responsive polymer with great potential for different applications, particularly in biomedical areas.

## Methods

### Materials

Oligo(ethylene glycol) diacrylate (OEGDA, Mn = 575) was purchased from Aladdin (Shanghai, China). Methacrylic acid (MAA) was from Tianjin Guangcheng Chemicals (Tianjin, China), with inhibitors removed by passing through basic Al_2_O_3_ (Sinopharm Chemical Regent Co. Ltd., Shanghai, China). Ammonium persulfate (APS) was supplied by Tianjin Hedong Hongyan Chemicals (Tianjin, China). The dialysis bags (MWCO 3500) were from Union Carbide Corporation (Shanghai, China).

### Preparation of P(OEGDA-MAA)

The copolymer of OEGDA and MAA, P(OEGDA-MAA), was prepared by precipitation polymerization in water. For a typical process, MAA (0.1834 g, 2.11 mmol) and OEGDA (0.8166 g, 1.43 mmol, molar ratio of OEGDA/MAA = 40/60) were added into 500 mL of deionized water pre-located in a glass flask of 1 L capacity. After nitrogen purging to remove oxygen, APS solution (10 wt%, 300 μL) was added. The flask was quickly sealed off and located into a water bath at 70 °C to start the polymerization for 4 h usually. The clear content in the bottle quickly became turbid after initiation, indicating the precipitation of the polymer. At the end of the polymerization, the reaction system was cooled down to room temperature, and the turbid emulsion-like mixture turned into a clear polymer dispersion, to which a certain amount of NaCl solution (2 M) with pH at 1.0, pre-adjusted using hydrochloric acid, was added. The addition of NaCl solution provoked the precipitation of the polymer, which was collected by centrifugation at 10,000 rpm, redispersed in water, and finally dialyzed against deionized water at room temperature for 72 h to remove NaCl.

### Characterization

^1^H NMR analysis of the polymers was performed on a 400-MHz NMR spectrometer (Avance III, Bruker, Switzerland) using dimethyl sulfoxide-*d*_6_ (DMSO-*d*_6_) as the solvent. The morphology of the dried polymer was observed using a scanning electron microscope (SEM, FEI Quanta Feg-250, US). OEGDA content of the polymer was determined using potentiometric titration as previously described [30]. A dispersion of the polymer in deionized water (1.0 mg/mL) was titrated to pH 11.5 with calibrated NaOH solution of about 0.1 M. pH of the dispersion was measured and recorded with a pH meter (Metrohm 808 Titrandio, Switzerland). The equivalence point was used to determine MAA content in the copolymer. VPTT of P(OEGDA-MAA) in a gastric dispersion (pH = 1.0, 150 mM NaCl) was measured as previously described [30]. VPTT of P(OEGDA-MAA) in its ethanol dispersion was determined by following the transmittance of the dispersion by a light of 565 nm using a spectrophotometer (UV3101PC, Shimadzu, Japan). The polymer dispersion was first heated to 70 °C, above the transition temperature, equilibrated for 5 min, and followed by cooling down from 65 to 10 °C at an interval of 5 °C with also an equilibrium time of 5 min at each temperature. The transmittance versus temperature was recorded, and the temperature, at which 50% of the maximal transmittance was reached, was taken as the VPTT. The size and size distribution of P(OEGDA-MAA) hydrogels in ethanol and in water were measured by dynamic light scattering (DLS, Nano-ZS, Malvern Instruments, UK).

## Results and Discussion

### Composition Analysis of P(OEGDA-MAA)

**Fig. 1:**
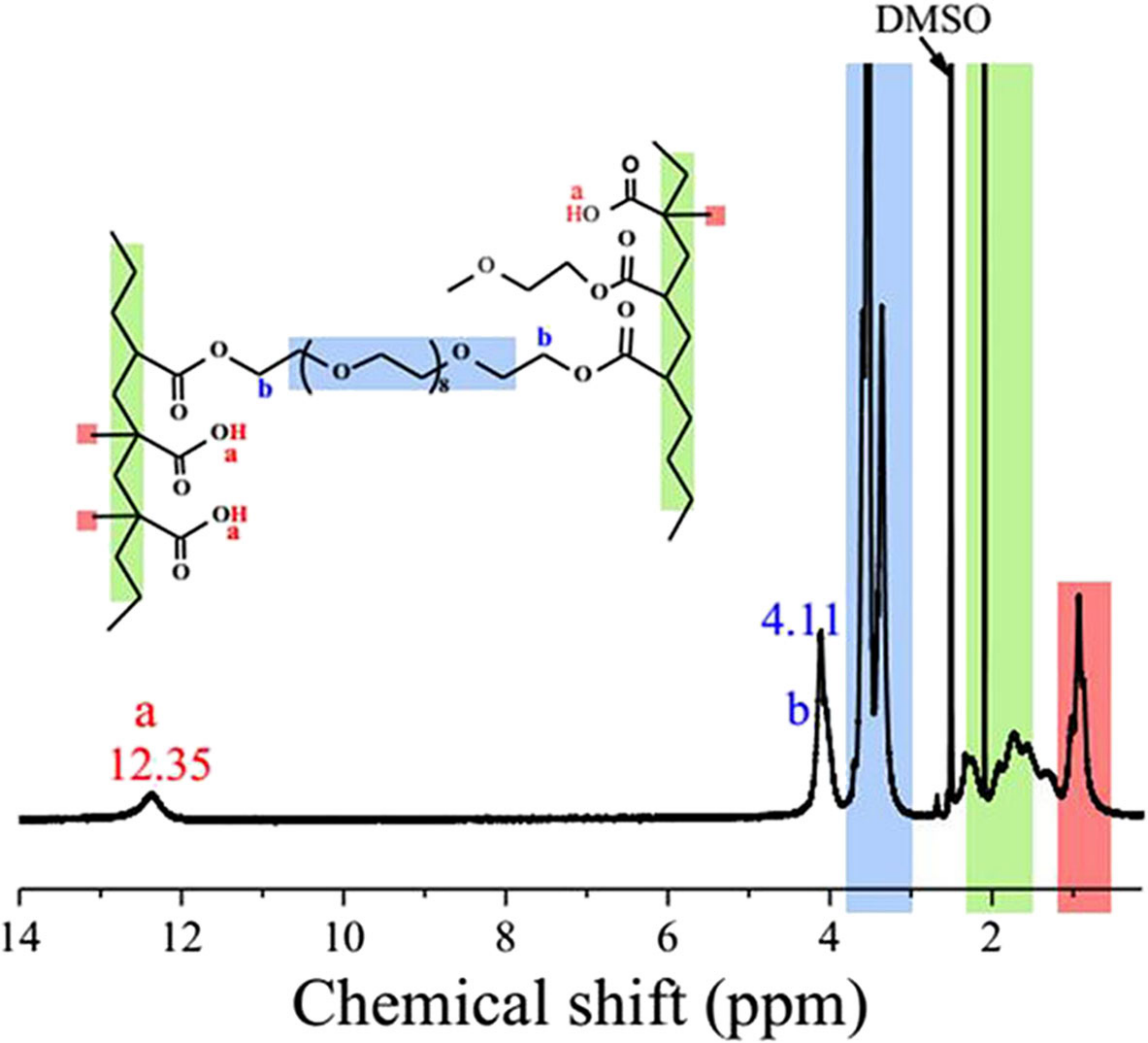
^1^H NMR spectrum of P(OEGDA-MAA)

It is notable that, in the reported preparations of diverse OEG-based thermoresponsive polymers, a great majority of OEG macromonomers is functionalized at one terminal only, and for most of which methacrylate is used as the terminal groups, whereas for the comonomers of low molecular weight, a variety of (meth)acrylates and acrylamide are used and MAA is rarely used [3, 13–17]. To prepare a novel type of OEG-based thermoresponsive polymers, a new type of OEG with both terminals functionalized with acrylate (OEGDA) was copolymerized in water with MAA, also a rarely used monomer (see Additional file 1: Figure S1). The polymerization was done with 40% of OEGDA in moles. The composition of the obtained P(OEGDA-MAA) was analyzed by ^1^H NMR (Fig. 1). The assignments of the peaks were carried out based on those of the monomers used (see Additional file 1: Figure S2) and on the reported polymers with similar structures [31]. The group of the peaks at chemical shift of 1.2 ppm or lower zone was assigned to the methyl protons in MAA unit in the polymer; the large and sharp peak at 2.0 ppm was assigned to the protons in the main chain consisting of MAA and acrylic segments, while the group of the large peaks appeared from 3.2 to 3.9 ppm was assigned to the protons of the ethylene of the backbone. The characteristic peaks, at chemical shifts of 12.35 and 4.11 ppm (Fig. 1) attributed to the carboxylic proton of MAA (Ha) and the oxyethylene ester protons (Hb) immediately adjacent to the acrylate units, were used to estimate the composition of the copolymer. OEGDA content in P(OEGDA-MAA) copolymer was calculated by Eq. 1:

1 $$ \left[\mathrm{OEGDA}\right]\ \mathrm{in}\ \mathrm{the}\ \mathrm{copolymer}\ \left(\mathrm{mol}\%\right)=\left({S}_{\mathrm{b}}/4\right)/\left({S}_{\mathrm{a}}+{S}_{\mathrm{b}}/4\right) $$

where *S*_a_ is the integrated surface area of the peak at 12.35 ppm representing the carboxylic protons and *S*_b_ is that of the peak at 4.11 ppm [30]. OEGDA content in the copolymer was thus obtained to be 40.9 mol%. This same OEGDA content was also estimated by potentiometric titration (see Additional file 1: Figure S3), which gave 38.8 mol% for OEGDA content. Compared with the ratio of the monomers used for the polymer preparation (40 mol% of OEGDA), one can conclude that the two monomers, OEGDA and MAA, were fully copolymerized.

### Morphology of P(OEGDA-MAA)

**Fig. 2:**
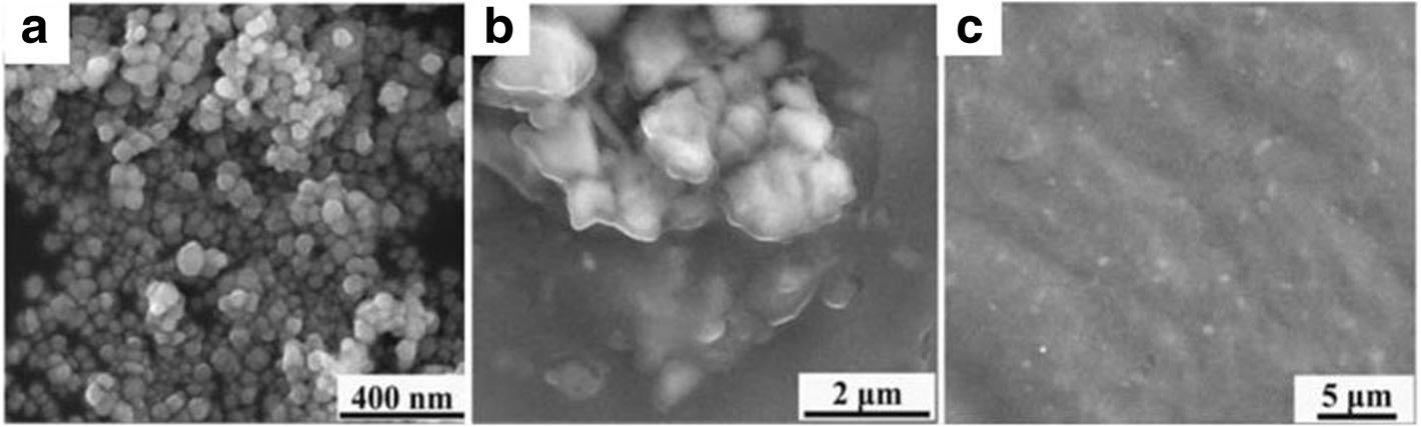
SEM images of P(OEGDA-MAA) obtained from their dispersion at room temperature at different concentration in water at 1 mg/mL (**a**) and in ethanol at 5 mg/mL (**b**) and 10 mg/mL (**c**)

**Fig. 3:**
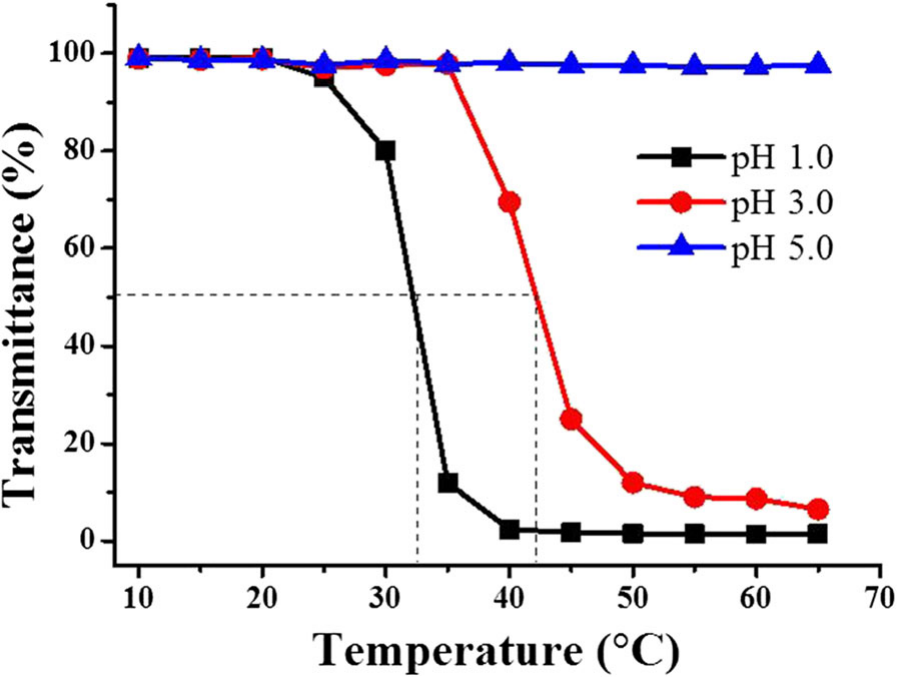
Dependence of light transmittance of P(OEGDA-MAA) dispersion (1.0 mg/mL, 150 mM NaCl) on temperature at different pH

To prepare the aqueous dispersion of P(OEGDA-MAA), the dried polymer hydrogel of known amount was dispersed in a given amount of water at room temperature, a clear solution-like dispersion was obtained by simple hand-shaking the vial, because this was done below the VPTT of the polymer in water, referred to as low-VPTT hereafter. For the dispersion of P(OEGDA-MAA) in ethanol, a known amount of the polymer was added into ethanol of known amount in a screw-cap glass bottle, pre-located in a water bath at 70 °C under magnetic stirring. Under gentle stirring, a quasi-clear dispersion was formed within 30 min to 1 h, depending on the polymer concentration. For all the dispersions in ethanol with different polymer concentration, they were kept heating at 70 °C for 1 h in order to get the equilibrated swelling for the polymer, followed by cooling down to room temperature, which turned the quasi-clear dispersions turbid, since this room temperature was far below the VPTT of the polymer in ethanol. The clear aqueous dispersion and the turbid suspension in ethanol of the polymer were taken out and dropped on the sample support for the morphology observation by SEM. Selected images are given in Fig. 2. It is seen that nano-sized hydrogel particles, with their number-average diameter of about 40 nm, were clearly observed for the sample prepared from the aqueous dispersion (1.0 mg/mL, Fig. 2a). It is notable that the polymerization was carried out at 70 °C, a temperature significantly above the low-VPTT of the polymer, in which polymer particles were formed. Though the polymer formed was not soluble in water, they must be highly hydrophilic knowing that they are mainly consisted of ethoxylate and methacrylic acid. Taking this into account, it is conceivable that the polymer would precipitate out in a later stage of the polymerization, as was observed that the clear solution became turbid around 30 min after the initiation, a much longer time than in conventional precipitation polymerization [32, 33]. In addition, the monomer concentration was very low (0.2 wt%), and the polymer formed, P(OEGDA-MAA), was crosslinked. All these combined, the formation of nanogel particles of very small size, about 40 nm at dried state, was not surprising. At room temperature in water, either by cooling down the polymerization system at end of the polymerization or by redispersing the dried polymer in water, these hydrogel particles are believed not to be truly dissolved at a molecular level because the polymer was crosslinked, they were instead largely swollen by water at such a large extent that the polymerization system looked like a veritable polymer solution with a transmittance of 100% (Fig. 3). In fact, a hydrodynamic particle size (*R*_h_, the radius) of about 12 nm was detected in this system at the end of the polymerization by DLS (Additional file 1: Figure S4). Compared to the size (diameter, 40 nm) of the particles at dry state (Fig. 2a), the DLS size of the hydrogel particles was even smaller, which suggests that the hydrogel particles must have aggregated during water evaporation in the SEM sample. This is easy to understand, since the hydrogel particles, with 100% of transmittance in its aqueous dispersion, must be largely swelled, and much smaller-sized particles at their dry state than the DLS size were anticipated if each of them had been kept independent and formed solid particles when the solvent was removed. Knowing that the crosslinked polymer was fully swelled, with their hydrophilic chain ends largely extended into the surrounding water phase, their hydrophilic chains and terminals ought to be interpenetrated to some extent. This made the whole dispersion highly homogeneous. The 100% transmittance of the dispersion was an indication of the homogeneity. Under this circumstance, some aggregation of the hydrogel particles was not unexpected with water evaporation; it should be in fact unavoidable.

As to the turbid mixture of P(OEGDA-MAA) in ethanol at room temperature, the sample was prepared in the same way, by dropping it on the sample support and the morphology observed under SEM. For the sample at 5 mg/mL concentration, it was found that the nanoparticles, observed in water as shown in Fig. 2a, disappeared (Fig. 2b). Instead, aggregated granules of the hydrogel polymer appeared, and the size of the granules was significantly larger than that of the particles obtained from their water dispersion (Fig. 2a). This is a strong indication that the deswelling was quite different for the two types of the dispersions.

It is most likely that the hydrogel polymer chains were more interpenetrated in ethanol above the high VPTT of UCST-type than they were in water below the low VPTT of LCST-type. This is very likely since ethanol is more similar to P(OEGDA-MAA) in the structure and hydrophilicity. By cooling down to room temperature, the original nanoparticles of the hydrogel polymers, formed in their synthesis and largely swelled above this UCST-type high VPTT, were stuck together and the granules or their aggregation were therefore observed. At a higher concentration (10 mg/mL, Fig. 2c) in ethanol, it seemed that the hydrogel polymer chains were further interpenetrated that a continuous hydrogel film was formed, with randomly dispersed small granules present.

### Low VPTT of P(OEGDA-MAA) Hydrogel in Water

This polymer, P(OEGDA-MAA) as synthesized above, was thermoresponsive (Fig. 3), and the responsiveness was also closely dependent on pH. A VPTT of 33 °C was detected in water at concentration of 1 mg/mL and pH 1.0, and this VPTT shifted to a higher temperature at 42 °C at pH 3, an obvious increase with increased pH. With pH further increased to 5, the polymer did not show any change in the light transmittance, i.e., the VPTT disappeared. These observations suggest that the presence of hydrogen ions was necessary for the hydrogel polymer to be thermoresponsive, quite different from most of the OEG-based responsive materials prepared with monomers free of carboxylic acid, which remain responsive at pH = 7 or higher [34]. It is known that a responsive polymer is solubilized, or swelled if crosslinked, at room temperature by different interactions between their chains and water molecules, i.e., their VPTT behavior can be regarded as the consequence of the competition between hydrophilic polymer-water interactions and hydrophobic polymer-polymer interactions [1, 2, 7, 16, 35]. Below this VPTT, the interactions between hydrophilic portion of the polymer and water are favored, water molecules are arranged around the polymer chains, establishing hydrogen bonds with the hydrophilic EG segments, leading to polymer solubilization if linear, and swelling when crosslinked, while above VPTT, this interaction of water-polymer is reduced, synchronously with increased interactions between the hydrophobic polymer themselves, leading to the dehydration of the polymer chains and their self-aggregation. For most of the OEG-based polymers without carboxylic moiety, there is no strong hydrogen bond donor in their chains but weak van der Waals interactions. The dependence of VPTT on pH is relatively moderate. However, the situation is dramatically changed when carboxylic groups are incorporated in the polymer chains, as in the present case for P(OEGDA-MAA). At low temperature (< VPTT) combined with low pH, below pKa (4.8) of the carboxylic acid [1, 3], the carboxylic groups are protonated and they may play the role of proton donor to the ether oxygen of the polymer to form hydrogen bonding, and such a complexation results in a sort of shrinkage of the hydrogel because the interaction of water molecules with the polymer chains is reduced this way, though the polymer chains remain hydrated and the polymer appears solubilized with high transmittance. At high pH, the carboxyl groups become ionized, leading to an electrostatic repulsion among the negatively charged polymer chains. This repulsion is much larger than all the interactions that can be achieved by the non-electrolyte polymer chains between themselves or with water, i.e., van der Waals and hydrogen bonding, and makes the polymer chains at their most extended state as possible: a full dissolution is achieved for the polymers not crosslinked, and the chains are largely extended with the polymer largely swelled for those chemically crosslinked [3]. In Fig. 3 at pH 5, the disappearance of VPTT was the case where a large number of the polymer chains were ionized, which generated by consequence a repulsion between the polymer chains, making them remain swelled even at a higher temperature, without the collapsed state observed. At a lower pH (pH 3, for example), there were less ionized carboxylic groups on a same polymer chain, which was less extended at a given temperature; the interactions of non-electrolyte polymer chains, such as van der Waals and hydrogen bonding, were gaining more importance, and a delayed VPTT was therefore detected at a higher temperature. Obviously, the lower is the pH; the lower is the VPTT, just as observed in Fig. 3. In addition, this is true only for polymer chains which turn negatively charged at high pH. For the polymers which become cationic at high pH (those with amine groups for instance), the opposite is true: VPTT appears at higher pH, and it disappears at lower pH [3, 36].

**Fig. 4:**
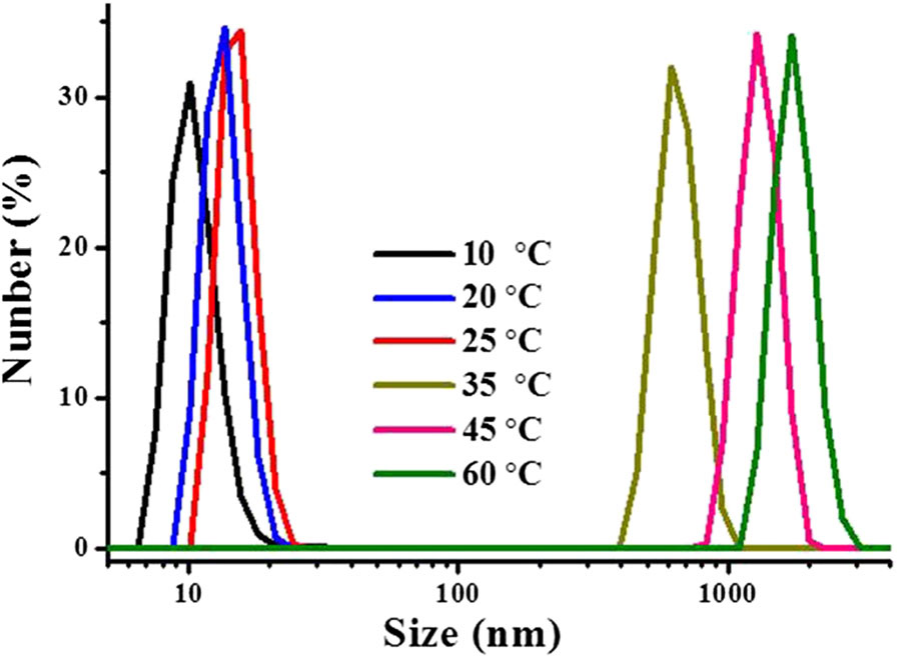
Diameter of thermoresponsive P(OEGDA-MAA) hydrogel in aqueous dispersion (1 mg/mL, pH 1.0) at different temperature

This VPTT transition was well demonstrated by the size variation of the hydrogel particles, determined by DLS at different temperature (Fig. 4) on an aqueous dispersion of P(OEGDA-MAA) at concentration of 1 mg/mL and pH 1.0. At a temperature below the VPTT (33 °C), very small particles were detected. The size was in slow and continuous increase with temperature increase, from about 15 nm at 10 °C to about 20 nm at 25 °C. An abrupt increase in the particle size was detected with the temperature increase across the VPTT, a diameter of the particles of about 600 nm was detected at 35 °C, just above the VPTT (33 °C); once the temperature is past the VPTT, another slow and gradual increase in the size of the dispersed polymer hydrogels was observed. It was quite obvious that the sizes detected here, about 600 nm at 35 °C and 1100 nm at 60 °C, were those of the aggregated hydrogel particles, in comparison with those obtained below the VPTT. Figure 4 revealed that the dehydration of the thermoresponsive polymer started much earlier before the VPTT; it was at the VPTT or in a temperature range very close to it that the dehydration was largely accelerated, which promoted the particles to shrink and to aggregate due to dehydration, and that a sharp decrease in transmittance was detected, as depicted in Fig. 3. The size variation of the hydrogel particles indicated also that the dehydration-caused aggregation of the hydrogel particles was still developing after the VPTT; it was not detectable simply by light transmittance because the decrease in the transmittance thereby was quite minimal in comparison to the high opacity at this state.

Besides the responsiveness dependence on pH as seen in Fig. 3, the thermoresponsive behavior of the material is also closely related to ionic strength, concentration, and the rate of heating/cooling. It has been shown by different authors that VPTT of OEG-based polymers is rather insensitive to concentration [14, 37]. However, Wu et al. reported, in a study on a copolymer of 2-(2-methoxyethoxy)ethyl methacrylate with oligo(ethylene glycol) methyl ether methacrylate, that a slight difference was observed and assumingly attributed to a higher concentration (10 wt%) than in other studies [16]. The influence of the polymer concentration on the VPTT was briefly investigated, with the concentration varied from 1.0 to 5.0 mg/mL (see Additional file 1: Figure S5). It was found that, in comparison with the VPTT observed at 33 °C for the hydrogel at concentration of 1.0 mg/mL, the VPTT was shifted to a lower temperature of 29 °C at concentration of 2.0 mg/mL, and to a further lower temperature of about 27 °C with the concentration increased to 5.0 mg/mL. This is simply owing to the concentration effect of the copolymer. In the present heating process, far below the VPTT, all or most of the polymer chains were independently and largely swelled in their dispersion; around VPTT, they started to aggregate, and after VPTT, all polymers were aggregated to form a heterogeneous suspension of the polymers in the solvent (water in the present case), leading to 0% light transmittance. VPTT is the indication of the time point where the polymer chains start to aggregate massively. It is easy to conceive that the polymer chains are easy to encounter each other to aggregate in a dispersion of higher concentration, and a shorter time is required to have a reduction in transmittance equivalent to that in a dilute dispersion.

### VPTT of P(OEGDA-MAA) Hydrogel in Ethanol

In contrast to LCST-type VPTT, studies on the polymers with UCST or UCST-type VPTT have been reported at a much lessened extent [38]. Polysulfobetaine [39] and poly(*N*-acrylolyglyciamide) [40–42] are the polymers reportedly with UCST in water, while polyacryamide [43] and poly(acetoacetoxyethyl methacrylate) [44] are reported to exhibit UCST in binary ethanol/water solvent mixture. Roth et al. demonstrated that poly[oligo(ethylene glycol) methyl ether methacrylate], (POEGMA), exhibited UCST in a large variety of aliphatic alcohols and that the UCST phase transition temperature was dependent on the structure, molecular weight, and the concentration of POEGMA, as well as the solvent, co-solvents [45]. P(NIPAM) was also reported to exhibit UCST behavior in ethanol/water mixture [46].

**Fig. 5:**
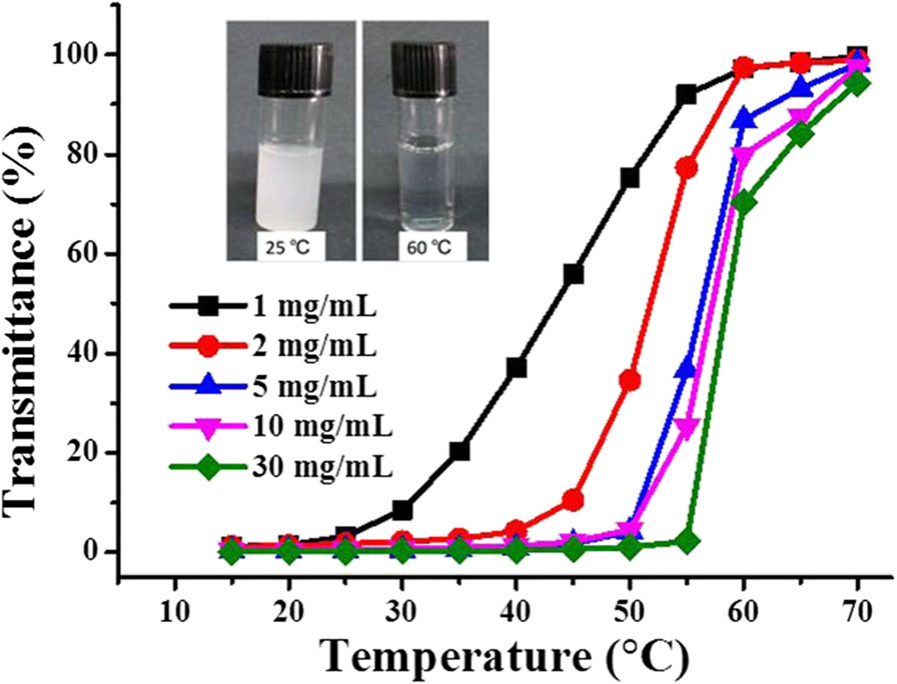
Transmittance of P(OEGDA-MAA) in ethanol with different concentration. The insets are the optical images of the hydrogels in ethanol (1 mg/mL) at 25 and 60 °C, respectively

Different from the test on the LCST-type VPTT in water, the study of UCST-type VPTT, of this material, was conducted in ethanol. The transmission was recorded from 70 °C to 15 °C with an equilibrium time of 5 min at each temperature (Fig. 5). When the temperature was at 70 °C or higher, the hydrogel-ethanol dispersion was homogeneous and transparent, with a transmittance of 100% for all the samples, except the one at the highest concentration of 30 mg/mL, of which the transmittance was about 95%. We believe a 100% transmittance would be reached with an extended equilibrium time. This high transmittance was an indication that the hydrogel polymer was largely swelled and that the dispersion looked like an authentic solution. With temperature decreasing, a decrease in transmittance was observed for all the samples and was clearly concentration related. With temperature decreased to 60 °C, the transmittance remained at 100% for the two samples with lower concentration (1 and 2 mg/mL), and a discernible decrease in transmittance was observed only when the temperature descended below 60 °C, whereas for the three samples with higher concentration (5, 10, and 30 mg/mL), an obvious decrease in the transmittance was detected starting from 70 °C, and the decrease was more significant for the sample of higher concentration. At 60 °C, the transmittance of the samples fell to 87, 80, and 70%, respectively, strictly in order of their concentration from low to high. This was exactly the same as that observed in the low VPTT in water, and readily understandable by the concentration effect. The only difference was that here it was concerned with ethanol instead of water, and by lowering the temperature, the intra- and intermolecular interactions of the hydrogel polymers became more important, these interactions overtook those between the polymer and ethanol. Ethanol molecules associated with polymer chains above the VPTT were gradually disassociated when the temperature was decreased, and the polymer chains started to self-aggregate when the temperature was close to the VPTT, leading to the decrease in light transmittance as shown in Fig. 5.

### Phase Transition Mechanism

**Fig. 6:**
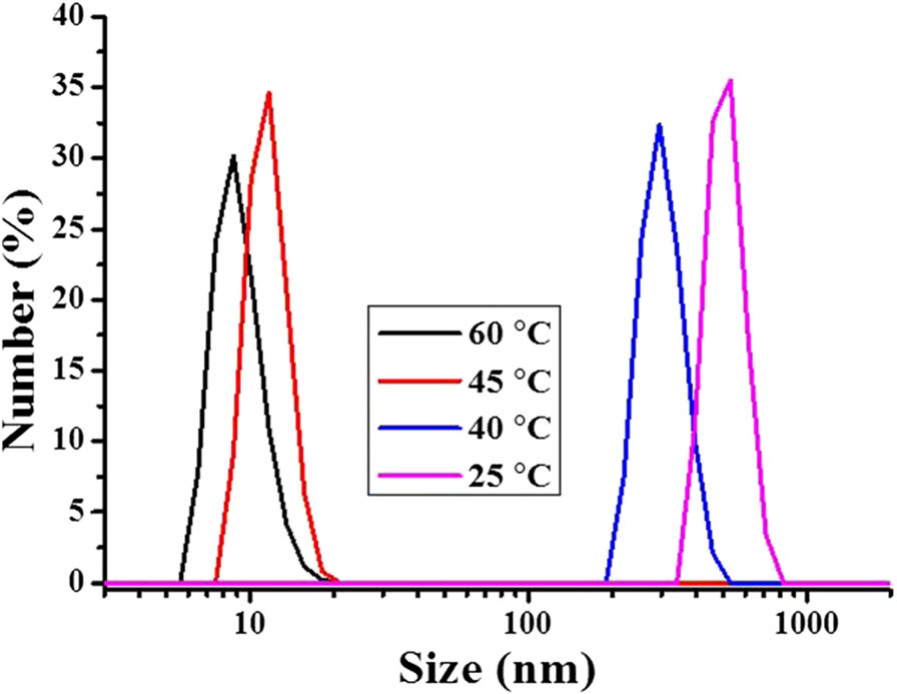
Diameter of P(OEGDA-MAA) hydrogels in ethanol (1 mg/mL) at different temperature

**Fig. 7:**
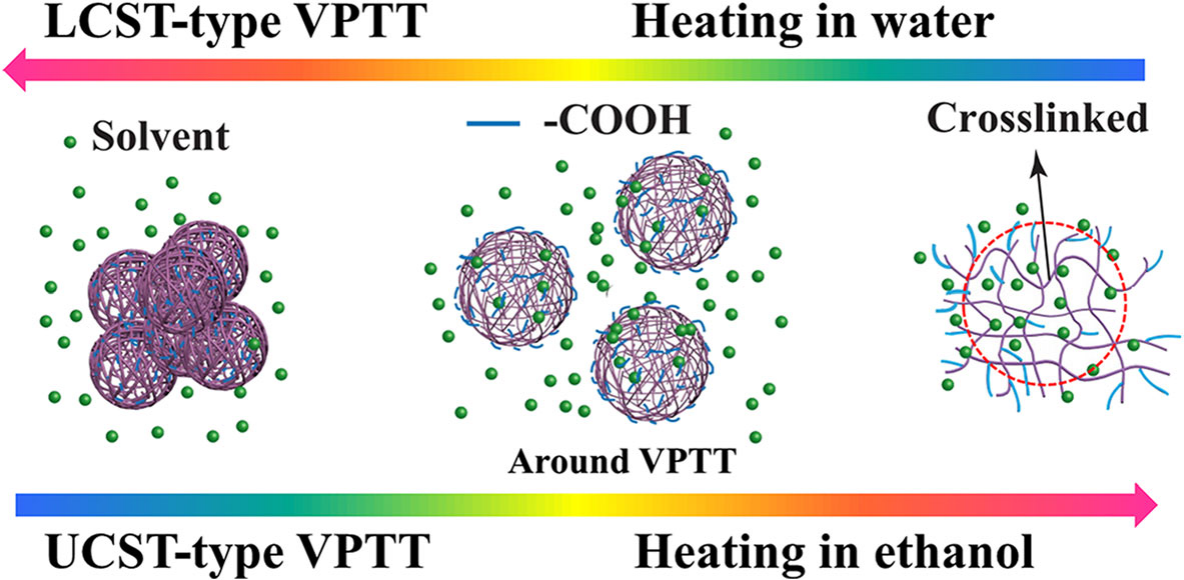
Schematic illustration of the VPTT mechanism for the UCST-type VPTT of P(OEGDA-MAA) in ethanol

To better understand the mechanism of this UCST-type VPTT, the size of the hydrogel particles at 1 mg/mL concentration was measured in the cooling process at different temperature (time) using DLS, exactly as that done for the LCST-type VPTT. The results are depicted in Fig. 6. A very small particle size, of about 8 nm in radius (*R*_h_), was obtained at 60 °C, though the sample looked homogeneous and transparent. The small size of the hydrogel particles was in agreement with the size of the nanogel in water below that low VPTT at 10 °C (diameter of 15 nm, Fig. 4), which may be an indication that the crosslinked hydrogel was largely swelled to a state just like being individually dissolved at a molecular level at such a low concentration, and the hydrophilic chain ends (mainly carboxylic groups) fully extended towards the surrounding solvent; the whole system was indeed homogeneous and appeared fully transparent. Given the amount of MAA used (60 mol%) and its high solubility in water, it is not excluded that some chain segments, and particularly chain ends consisting of only MAA, were well present in the polymers. These chain ends would be the most exposed towards the surrounding solvent in their fully swelled state, i.e., towards water below the LCST-type VPTT and towards ethanol above the UCST-type VPTT. Owing to the limited number of the hydrophilic chain ends and their high hydration, the size of the hydrogel particles detected might be only the size of the crosslinked core, as shown by the encircled area in Fig. 7. In fact, hydrogel particles with their *R*_h_ around 10 nm, determined by DLS, were often reported. Diverse examples are available, such as polyacrylamide above its UCST in aqueous alcohol solutions [43] and 4-propoxyazobenzene-terminated PNIPAM in aqueous solution below its LCST [47]. And *R*_h_ as small as 2 to 3 nm was reported for an OEG-based responsive polymer below its LCST [48]. For the present polymer, by decreasing the temperature from 60 to 45 °C, very close to the VPTT, the *R*_h_ of the hydrogel particle size slightly increased to about 7 nm. This increase might be caused by the collapse of the hydrophilic chain ends, which were extended into ethanol at temperature above the UCST-type VPTT as discussed above. At this stage, the crosslinked core of the hydrogel particles was largely shrunk because the ethanol molecules were squeezed out of the polymer inner, owing to a decrease in the interaction of the polymer chains with ethanol molecules by decreasing the temperature, accompanied by an increase in the interaction of the polymer chains between themselves. By further decreasing the temperature to 40 °C just across the UCST (43 °C), the radius of the hydrogel particles abruptly increased to a significantly large size of 150 nm, very similarly to what observed for the aqueous dispersion shown in Fig. 4 when the temperature was increased across the LCST-type VPTT. This size here is no more that of a single crosslinked nanogel as above but rather that of the aggregate of the nanogel particles, as illustrated in Fig. 7. The radius of the particle aggregates further grew to 270 nm with the temperature further decreased to 25 °C. This suggests that, before reaching the UCST-type VPTT, the transmittance decrease was due to mainly the change in polymer chain conformation, i.e., the hydrophilic chains (ethylene oxide segments, and particularly carboxylic groups) were changed from their full extension in the solvent to coils on the surface of the individual hydrogel polymer particles with a crosslinked core for each, whereas the transmittance decrease around the VPTT was caused by self-aggregation of the nanogel particles (unimers), in accordance with further disassociation of ethanol molecules from the polymer chains. This has been named as a two-step process including dehydration of the particles (unimers) and their aggregation [16, 35, 37, 49]. In characterization of LCST or VPTT by varying temperature, the disassociation of the hydrophilic chains from the solvent molecules leads to the formation of a core-shell structure, with the hydrophobic polymer chains as the core and the dehydrated chains as the shell, this mechanism is also described as formation of core-shell micelles followed by their aggregation [16, 37]. A schematic illustration is given in Fig. 7 for this mechanism.

## Conclusions

A novel responsive polymer based on OEG, P(OEGDA-MAA), is prepared through precipitation copolymerization of OEGDA with MAA. The polymer prepared with 40 mol% of OEGDA was chemically crosslinked and showed a distinct LCST-type VPTT of 33 °C in water at pH 1.0 and concentration of 1.0 mg/mL. This VPTT was closely concentration and pH dependent. It shifted towards lower temperature with increased concentration, whereas a shift towards higher temperature was observed with increased pH, and the VPTT completely disappeared at pH 5. This same polymer exhibited also a UCST-type VPTT in ethanol at 43 °C, which was equally concentration dependent. The size evolution of the hydrogel particles versus temperature was measured for the two types of dispersions across their VPTT. For the LCST-type VPTT in water, a slight size increase was detected with increased temperature as long as the temperature was below this VPTT; and a dramatic size increase was observed once the temperature was increased to above this LCST-type VPTT. For the UCST-type VPTT in ethanol, the opposite was observed, i.e., a slight size increase with decreased temperature as long as the temperature was above this VPTT, and a dramatic size increase once the temperature was lowered below the UCST-type VPTT. These results suggest that the responsiveness of the polymer follows a two-step process, including a transition of polymer chain conformation from extended status to coil-form due to the dehydration of the hydrophilic chains from the largely swelled state, followed by an aggregation of the individual particles. This work provides therefore a novel type of candidate materials for potential applications in biomedical fields.

## Additional file

Additional file 1: **Figure S1.** Illustration of the synthesis of thermoresponsive P(OEGDA-MAA). **Figure S2.** ^1^H NMR spectra of the monomers, oligo(ethylene glycol) diacrylate and methacrylic acid, used in the synthesis of thermoresponsive P(OEGDA-MAA). **Figure S3.** Potentiometric titration of thermoresponsive P(OEGDA-MAA). **Figure S4.** DLS diameter of P(OEGDA-MAA) hydrogel in water (1 mg/mL) at room temperature. **Figure S5.** Dependence of light transmittance on increasing temperature for aqueous dispersion of P(OEGDA-MAA) of different concentration (pH 1.0, 150 mM NaCl). The insets are the photos of the hydrogel at concentration of 1.0 mg/mL, taken at 25 and 60 °C, respectively. (PDF 167 kb)

## Abbreviations

APS: Ammonium persulfate
ATRP: Atom transfer radical polymerization
DLS: Dynamic light scattering
EG: Ethylene glycol
LCST: Lower critical solution temperature
MAA: Methacrylic acid
OEG: Oligo(ethylene glycol)
OEGDA: Oligo(ethylene glycol) diacrylate
P(OEGDA-MAA): Poly(oligo(ethylene glycol) diacrylate-methacrylic acid)
PEG: Poly(ethylene glycol)
PNIPAM: P(*N*-isopropylacrylamide)
POEGMA: Poly[(oligo(ethylene glycol) methyl ether methacrylate]
RAFT: Reversible addition-fragmentation chain transfer polymerization
*R*_h_: Hydrodynamic particle radius
UCST: Upper critical solution temperature
VPTT: Volume phase transition temperature

## References

[1] Ferreira NN, Ferreira LMB, Cardoso VMO, Boni FI, Souza ALR, Gremiao MPD (2018). Recent advances in smart hydrogels for biomedical applications: from self-assembly to functional approaches. Eur Polym J.

[2] Vermonden T, Censi R, Hennink W (2012). Hydrogels for protein delivery. Chem Rev.

[3] Etchenausia L, Khoukh A, Lejeune ED, Save M (2017). RAFT/MADIX emulsion copolymerization of vinyl acetate and N-vinylcaprolactam: towards waterborne physically crosslinked thermoresponsive particles. Polym Chem.

[4] Miladinovic ZR, Micic M, Mrakovic A, Suljovrujic E (2018). Smart hydrogels with ethylene glycol propylene glycol pendant chains. J Polym Res.

[5] Chang B, Chen D, Wang Y, Chen Y, Jiao Y, Sha X, Yang W (2013). Bioresponsive controlled drug release based on mesoporous silica nanoparticles coated with reductively sheddable polymer shell. Chem Mater.

[6] Samchenko Y, Uilberg Z, Korotych O (2011). Multipurpose smart hydrogel systems. Adv Colloid Interf Sci.

[7] Xu SF, Lu HZ, Zheng XW, Chen LX (2013). Stimuli-responsive molecularly imprinted polymers: versatile functional materials. J Mater Chem C.

[8] Bae YH, Okano T, Hsu R, Kim SW (1987). Thermo-sensitive polymers as on-off switches for drug release. Markomol Chem Rapid Commun.

[9] Roy D, Brooks WLA, Sumerlin BS (2013). New directions in thermoresponsive polymers. Chem Soc Rev.

[10] Yang Z, Layani M, Wang S, Hu P, Ke Y, Magdassi S, Yi L (2018). Fully printed flexible smart hydrogels. Adv Funct Mater.

[11] Xia Y, Yin XC, Burke NAD, Stöver HDH (2005). Thermal response of narrow-disperse poly(N-isopropylacrylamide) prepared by atom transfer radical polymerization. Macromolecules.

[12] Zhu X, Gu X, Zhang L, Kong XZ (2012). Preparation and characterization of nanosized P(NIPAM-MBA) hydrogel particles and adsorption of bovine serum albumin on their surface. Nanoscale Res Lett.

[13] Sun S, Wu P (2013). Role of water/methanol clustering dynamics on thermosensitivity of poly(N-isopropylacrylamide) from spectral and calorimetric insights. Macromolecules.

[14] Lutz JF, Akdemir Ö, Hoth A (2006). Point by point comparison of two thermosensitive polymers exhibiting a similar LCST: is the age of poly (NIPAM) over?. J Am Chem Soc.

[15] Lutz JF (2011). Thermo-switchable materials prepared using the OEGMA-platform. Adv Mater.

[16] Zhang B, Tang H, Wu P (2014). In depth analysis on the unusual multistep aggregation process of oligo(ethylene glycol) methacrylate-based polymers in water. Macromolecules.

[17] Badi N (2017). PEG-based thermoresponsive polymer systems. Prog Polym Sci.

[18] Saeki S, Kuwahara N, Nakata M, Kaneko M (1976). Upper and lower critical solution temperatures in poly(ethylene glycol) solutions. Polymer.

[19] Ishizone T, Seki A, Hagiwara M, Han S (2008). Anionic polymerizations of oligo(ethylene glycol) alkyl ether methacrylates: effect of side chain length and alkyl group of side chain on cloud point in water. Macromolecules.

[20] Sugihara S, Hashimoto K, Matsumoto Y, Kanaoka S, Aoshima S (2003). Thermosensitive polyalcohols: synthesis via living cationic polymerization of vinyl ethers with a silyloxy group. J Polym Sci Part A: Polym Chem.

[21] Vamvakaki M, Billingham NC, Armes SP (1999). Synthesis of water-soluble statistical copolymers and terpolymers containing pendent oligo(ethylene glycol derivatives). Polymer.

[22] Hadjiyannakou SC, Vamvakaki M, Patrickios CS (2004). Synthesis, characterization and evaluation of amphiphilic diblock copolymer emulsifiers based on methoxy hexa(ethylene glycol) methacrylate and benzyl methacrylate. Polymer.

[23] Driva P, Bexis P, Pitsikalis M (2011). Radical copolymerization of 2-vinyl pyridine and oligo(ethylene glycol) methyl ether methacrylates: monomer reactivity ratios and thermal properties. Eur Polym J.

[24] Zhu W, Nese A, Matyjaszewski K (2011). Thermoresponsive star triblock copolymers by combination of ROP and ATRP: from micelles to hydrogels. J Polym Sci Part A: Polym Chem.

[25] De P, Sumerlin BS (2013). Precision control of temperature response by copolymerization of di(ethylene glycol) acrylate and an acrylamide comonomer. Macromol Chem Phys.

[26] Yeniad B, Ryskulova K, Fournier D, Lyskawa J, Cooke G, Woisel P, Hoogenboom R (2016). Complexation of thermoresponsive dialkoxynaphthalene end-functionalized poly(oligoethylene glycol acrylate)s with CBPQT^4+^ in water. Polym Chem.

[27] Becer CR, Kokado K, Weber C, Can A, Chujo Y (2010). Metal-free synthesis of responsive polymers: cloud point tuning by controlled click reaction. J Polym Sci Part A: Polym Chem.

[28] Soeriyadi AH, Li GZ, Slavin S, Jones MW, Amos CM, Becer CR, Whittaker MR, Haddleton DM, Boyer C, Davis TP (2011). Synthesis and modification of thermoresponsive poly(oligo(ethylene glycol) methacrylate) via catalytic chain transfer polymerization and thiol-ene Michael addition. Polym Chem.

[29] Sakai N, Jin M, Sato S, Satoh T, Kakuchi T (2014). Synthesis of water-soluble polyisocyanates with the oligo(ethylene glycol) side-chain as new thermoresponsive polymers. Polym Chem.

[30] Cao H, Wang Q, Li M, Chen Z (2015). Synthesis of stimuli-responsive poly(ethylene glycol) diacrylate/methacrylic acid-based nanogels and their application as drug delivery vehicle. Colloid Polym Sci.

[31] Can A, Zhang QL, Rudolph T, Schacher FH, Gohy JF, Schubert US, Hoogenboom R (2015). Schizophrenic thermoresponsive block copolymer micelles based on LCST and UCST behavior in ethanol–water mixtures. Eur Polym J.

[32] Kong XZ, Gu X, Zhu X, Zhang L (2009). Precipitation polymerization in ethanol and ethanol/water to prepare uniform microspheres of poly(TMPTA-styrene). Macromol Rapid Commun.

[33] Jiang X, Zhu X, Kong XZ (2012). A facile route to preparation of uniform polymer microspheres by quiescent polymerization with reactor standing still without any stirring. Chem Eng J.

[34] Medel S, Garcia JM, Garrido L, Quijada-garrido I, Paris RT (2011). Thermo- and pH-responsive gradient and block copolymers based on 2-(2-methoxyethoxy)ethyl methacrylate synthesized via atom transfer radical polymerization and the formation of thermoresponsive surfaces. J Polym Sci: Part A: Polym Chem.

[35] Sun S, Wu P (2013). On the thermally reversible dynamic hydration behavior of OEG methacrylate-based polymers in water. Macromolecules.

[36] Moon JR, Park YH, Kim J (2009). Synthesis and characterization of novel thermo- and pH-responsive copolymers based on amphiphilic polyaspartamides. J Appl Polym Sci.

[37] Peng B, Grishkewich N, Yao Z, Han X, Liu H, Tam KC (2012). Self-assembly behavior of thermoresponsive oligo(ethylene glycol) methacrylates random copolymer. ACS Macro Lett.

[38] Zhu M, Xu Y, Ge C, Ling Y, Tang H (2016). Synthesis and UCST-type phase behaviour of OEGylated poly(γ-benzyl-L-glutamate) in organic media. J Polym Sci, Part A: Polym Chem.

[39] Kudaibergenov S, Jaeger W, Laschewsky A (2006). Polymeric betaines: synthesis, characterization, and application. Adv Polym Sci.

[40] Glatel S, Laschewsky A, Lutz JF (2011). Well-defined uncharged polymers with a sharp UCST in water and in physiological milieu. Macromolecules.

[41] Liu FY, Seuring J, Agarwal S (2012). Controlled radical polymerization of N-acryloylglycinamide and UCST-type phase transition of the polymers. J Polym Sci, Part A: Polym Chem.

[42] Seuring J, Bayer FM, Huber K, Agarwal S (2012). Upper critical solution temperature of poly(N-acryloyl glycinamide) in water: a concealed property. Macromolecules.

[43] Asadujjaman A, de Oliveria TE, Mukherji D, Bertin A (2018). Polyacrylamide “revisited”: UCST-type reversible thermoresponsive properties in aqueous alcoholic solutions. Soft Matter.

[44] Boyko V, Lu Y, Richter A, Pich A (2003). Preparation and characterization of acetoacetoxyethyl methacrylate-based gels. Macromol Chem Phys.

[45] Roth PJ, Jochum FD, Theato P (2011). UCST-type behavior of poly[oligo(ethylene glycol) methyl ether methacrylate] (POEGMA) in aliphatic alcohols: solvent, co-solvent, molecular weight, and end group dependences. Soft Matter.

[46] Liu L, Wang T, Liu C, Lin K, Liu G, Zhang G (2013). Specific anion effect in water-nonaqueous solvent mixtures: interplay of the interactions between anion solvent, and polymer. J Phys Chem B.

[47] Xue X, Yang J, Huang W, Yang H, Jiang B, Li F, Jiang Y (2015). Dual thermo- and light-responsive nanorods from self-assembly of the 4-propoxyazobenzene-terminated poly(NIPAM) in aqueous solution. Polymer.

[48] Lutz JF, Weichenhan K, Akdemir Ö, Hoth A (2007). About the phase transition in aqueous solutions of thermoresponsive copolymers and hydrogels based on 2-(2-methoxyethoxy)ethyl methacrylate and oligo(ethylene glycol) methacrylate. Macromolecules.

[49] Lutz JF (2008). Polymerization of oligo(ethylene glycol) (meth)acrylates: toward new generations of smart biocompatible materials. J Polym Sci, Part A: Polym Chem.

