# Supplementary material for: Preparation of Thermoresponsive Polymer Nanogels of Oligo(Ethylene Glycol) Diacrylate-Methacrylic Acid and Their Property Characterization
Source: Nanoscale Res Lett. 2018 Jul 13;13:209. doi: 10.1186/s11671-018-2610-6 (PMC6045526; doi:10.1186/s11671-018-2610-6)
Supplement: Supplementary file 1 — Figure S1. Illustration of the synthesis of thermoresponsive P(OEGDA-MAA). Figure S2. 1H NMR spectra of the monomers, oligo(ethylene glycol) diacrylate and methacrylic acid, used in the synthesis of thermoresponsive P(OEGDA-MAA). Figure S3. Potentiometric titration of thermoresponsive P(OEGDA-MAA). Figure S4. DLS diameter of P(OEGDA-MAA) hydrogel in water (1 mg/mL) at room temperature. Figure S5. Dependence of light transmittance on increasing temperature for aqueous dispersion of P(OEGDA-MAA) of different concentration (pH 1.0, 150 mM NaCl). The insets are the photos of the hydrogel at concentration of 1.0 mg/mL, taken at 25 and 60 °C, respectively. (PDF 167 kb) [file 11671_2018_2610_MOESM1_ESM.pdf]

Preparation of thermoresponsive polymer nanogels of oligo(ethylene glycol) diacrylate-methacrylic acid and their property characterization

Hongyan Cao, Fenghao Guo, Zhiyong Chen* and Xiang Zheng Kong*

College of Chemistry and Chemical Engineering, University of Jinan, Jinan 250022, China

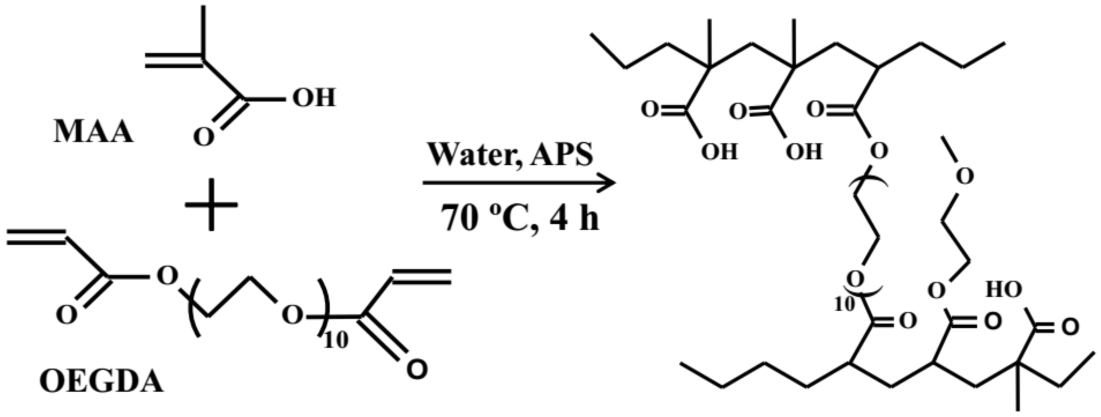

Figure S1. Illustration of the synthesis of thermoresponsive P(OEGDA-MAA)

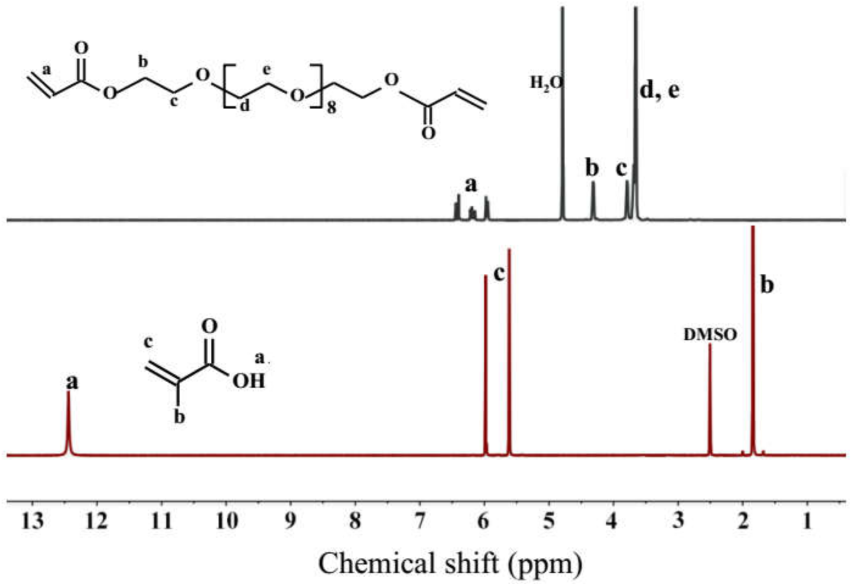

Figure S2. ^{1}H NMR spectra of the monomers, oligo(ethylene glycol) diacrylate and methacrylic acid, used in the synthesis of thermoresponsive P(OEGDA-MAA)

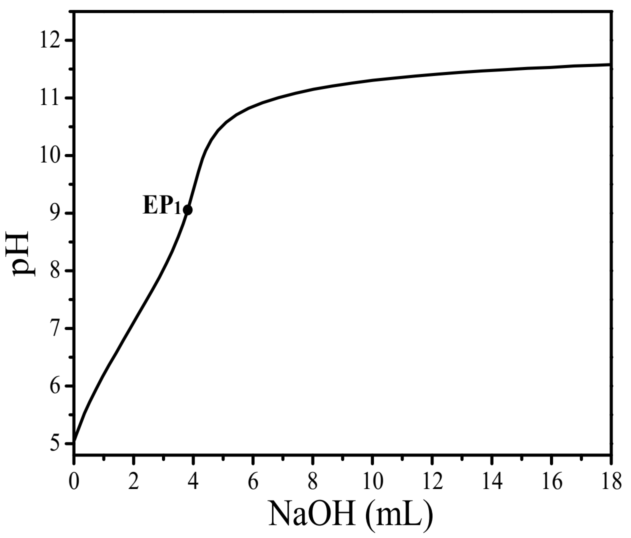

Figure S3. Potentiometric titration of thermoresponsive P(OEGDA-MAA)

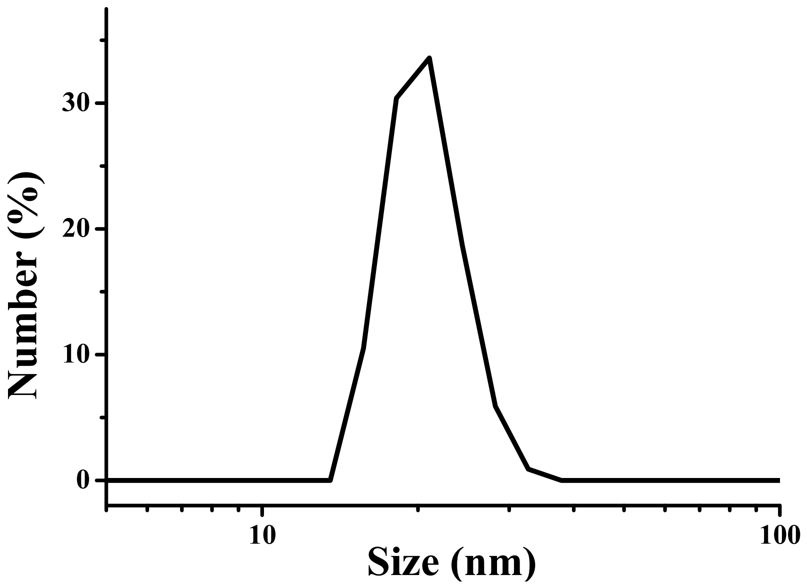

Figure S4. DLS diameter of P(OEGDA-MAA) hydrogel in water (1 mg/mL) at room temperature

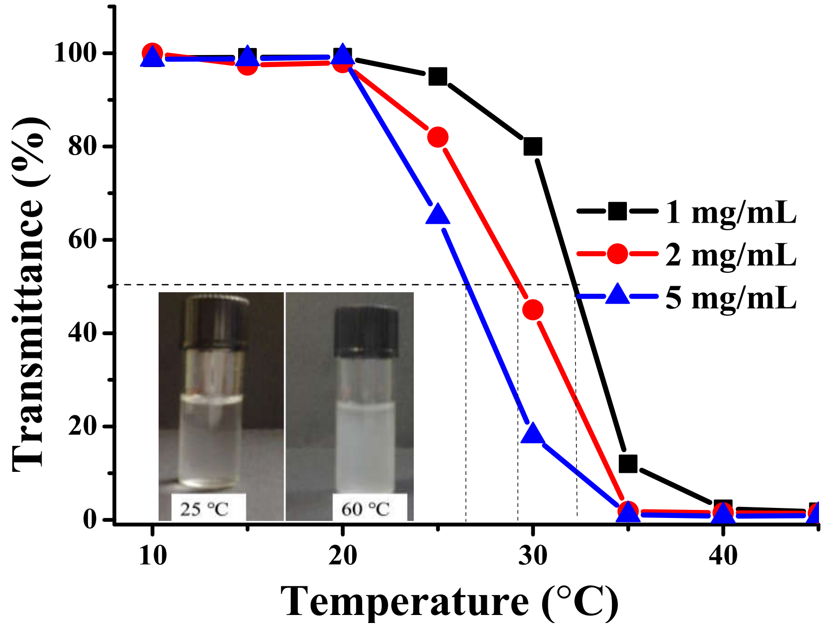

Figure S5. Dependence of light transmittance on increasing temperature for aqueous dispersion of P(OEGDA-MAA) of different concentration (pH 1.0, 150 mM NaCl). The insets are the photos of the hydrogel at concentration of 1.0 mg/mL, taken at 25 ºC and 60 ºC, respectively.
